# Trend-Conditioned Residual Learning for Early Fault Warning in Nonstationary Multi-Sensor Oil Monitoring

**DOI:** 10.3390/s26123779

**Published:** 2026-06-13

**Authors:** Huaqing Li, Yongxu Chen, Yitian Wang, Changlin Wu

**Affiliations:** 1 School of Naval Architecture, Ocean and Energy Power Engineering, Wuhan University of Technology, Wuhan 430063, China; huaqingli.work@whut.edu.cn; 2 School of Transportation and Logistics Engineering, Wuhan University of Technology, Wuhan 430063, China; cyx_1005@whut.edu.cn; 3 School of Optoelectronic Engineering, Xi’an Technological University, Xi’an 710021, China; wangyitian@st.xatu.edu.cn; 4 School of Mechanical and Electrical Engineering, Huainan Normal University, Huainan 232001, China

**Keywords:** fault diagnosis, condition monitoring, multi-sensor oil monitoring, early fault warning, anomaly detection, diffusion models, dynamic risk band

## Abstract

Lubricating oil monitoring provides continuous health information for early fault warning and maintenance decision-making in industrial gas turbines. However, real-world multi-sensor monitoring streams exhibit pronounced nonstationary thermodynamic drifts that often obscure subtle high-frequency residuals containing critical incipient degradation signatures. Prevailing data-driven monitoring models typically struggle to separate these macroscopic trends from stochastic wear-related fluctuations, and their restrictive distributional assumptions are often inadequate for the heteroscedastic and heavy-tailed nature of industrial residuals. To address these challenges, this study proposes ResAD-Net, a framework for early fault warning in nonstationary multi-sensor oil monitoring that combines trend–residual decoupling, trend-conditioned residual modeling, and residual-domain dependency learning. Specifically, a signal trend–residual decoupling strategy is adopted to separate slowly varying operational trends from stochastic residual fluctuations captured by the sensors, thereby exposing residual information that is more sensitive to incipient degradation. On this basis, a trend-conditioned diffusion model is introduced to characterize state-dependent, skewed residual distributions and generate residual sample ensembles for nonstationary monitoring. Meanwhile, a graph-based variational autoencoder is employed to learn latent intersensor dependency structures from the residual domain, providing diagnostic cues for temporal risk evolution analysis and sensor-level inspection. Experiments on a real-world industrial oil-monitoring record show that the proposed framework achieves an average F1-score of 0.985 with no observed false positives in the predefined pre-alarm reference interval of the finite test set. In addition to accurate anomaly detection, ResAD-Net captures early residual distributional shifts before clear macroscopic deviations emerge and provides diagnostic association cues for interpreting oil-monitoring changes around the system-level alarm.

## 1. Introduction

Industrial gas turbines are increasingly operated under flexible and deep peak-shaving conditions, which subject critical components to repeated thermal and mechanical stress cycles [1]. In such systems, lubricating oil monitoring provides an important basis for predictive maintenance because it can reveal incipient wear-related abnormalities earlier than many conventional condition indicators [2]. It also offers a practical source of condition information for anomaly identification in industrial oil-monitoring scenarios [3], where heterogeneous sensors are deployed to capture various fluid properties. In practice, however, these multi-sensor data are strongly nonstationary and exhibit pronounced thermodynamic drift together with complex cross-variable coupling [3]. Under such conditions, weak degradation-sensitive fluctuations can be obscured by dominant baseline evolution, complicating reliable early fault warning.

The main difficulty arises from the coexistence of two signal components with distinct physical meanings in the sensor readings: a slowly varying macroscopic trend associated with operating-state evolution, and a stochastic residual component that is often more sensitive to incipient degradation. Learning-based models trained with pointwise objectives such as mean squared error generally fit low-frequency structures more readily than subtle residual fluctuations, a tendency consistent with the spectral bias reported for deep models [4,5]. In multi-sensor anomaly detection, this preference may reduce sensitivity to weak early-stage signatures [6]. As a result, early degradation-related changes can remain difficult to identify during the pre-alarm evolution stage even when residual behavior has already begun to shift [7,8]. Such distributional changes may precede obvious macroscopic excursions and are therefore relevant to early warning [9]. This challenge is further compounded by the widespread use of fixed alarm thresholds, whose amplitude-centered logic is often less sensitive to gradual distributional deterioration [7].

A natural way to address this issue is to separate the slowly varying background from the more informative stochastic component. Traditional decomposition methods can assist in this process [10,11,12], but preserving diagnostically meaningful structural transitions while avoiding oversmoothing or artificial artifacts remains difficult for nonstationary industrial signals [13]. Early warning also requires an operating-state trend reference that can characterize expected macroscopic evolution under changing operating conditions. This has motivated the development of baseline forecasting models, including transformer-based architectures [14,15,16], lightweight linear or statistical forecasting methods [17,18], and recurrent neural networks [19]. In nonstationary settings, greater model complexity does not necessarily lead to a more reliable trend reference, and simpler formulations may provide favorable robustness and efficiency [17]. At the same time, channel-independent modeling strategies may weaken endogenous thermodynamic coupling and thus reduce the physical plausibility of multivariate baseline evolution [20,21].

The residual component after decoupling is equally important because it retains stochastic degradation information that is often masked in the raw signal. In industrial environments, such residuals are often irregular, heteroscedastic, skewed, and heavy-tailed, making deterministic regression or standard Gaussian assumptions insufficient for state-dependent uncertainty modeling [22,23]. Diffusion-based generative models offer a flexible way to describe complex time-series distributions [24,25,26,27], but most existing studies focus on probabilistic forecasting or imputation rather than on residual generation adapted to changing operating states [25,26,27].

Related industrial anomaly-detection and prognostics and health management (PHM) studies have explored reconstruction-based, forecasting-based, and graph-based paradigms. These include autoencoder-based fault detection [28], unsupervised generative gearbox anomaly detection [29], adversarial reconstruction for multivariate time-series anomalies [30], predictive-maintenance studies that emphasize continuous condition indicators for Industry 4.0 maintenance decisions [31], and residual-error variational-autoencoder-based approaches [32]. Forecasting- and graph-based methods further model deviations from expected temporal evolution and intersensor dependencies [17,32,33,34]. However, many current methods still operate on mixed signals or rely on static and weakly adaptive structures [35,36], which can limit early warning and diagnostic interpretation in strongly coupled industrial scenarios [37,38,39].

For sensor-enabled fault diagnosis and health management, the key challenge is therefore not only to acquire continuous monitoring signals but also to transform nonstationary sensor streams into robust and interpretable warning evidence. This requirement is particularly important in oil-monitoring applications, where early degradation information is often embedded in weak residual fluctuations rather than in obvious macroscopic excursions. A practical diagnostic framework should therefore provide an unsupervised normal-pattern reference, characterize residual uncertainty under changing operating states, and offer sensor-level cues for maintenance investigation and diagnostic prioritization.

The primary contributions of this study are summarized as follows:

- We propose ResAD-Net as a residual-centered early-warning framework for nonstationary multi-sensor oil monitoring, shifting the analysis from mixed-signal anomaly detection to trend–residual separated warning evidence.
- We develop a trend-conditioned residual modeling strategy based on diffusion generation to characterize state-dependent, heteroscedastic, and non-Gaussian residual uncertainty and to generate residual sample ensembles for downstream score-domain risk-band construction beyond rigid deterministic thresholds and fixed Gaussian assumptions.
- We incorporate residual-domain dependency learning through a dual-stream Graph Variational Autoencoder (GVAE), allowing the framework to support robust anomaly detection, temporal risk evolution analysis, and maintenance-oriented sensor-level diagnostic cues.

The remainder of this paper is organized as follows. Section 2 details the ResAD-Net methodology, including trend–residual decoupling, deterministic trend forecasting, residual generation, and risk-aware anomaly scoring. Section 3 describes the industrial oil-monitoring dataset and evaluation protocol. Section 4 reports the experimental results and diagnostic analysis. Section 5 concludes the study.

## 2. Methodology

### 2.1. Overview of the Framework

**Figure 1:**
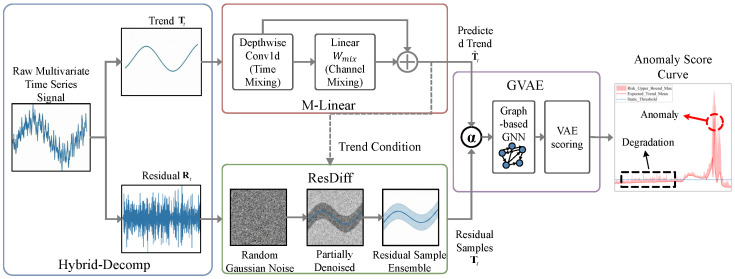
Overall architecture of the proposed ResAD-Net.

ResAD-Net addresses anomaly detection in nonstationary oil monitoring by explicitly decoupling the raw multi-sensor signal $X_{t}$ into a deterministic trend $T_{t}$ and a stochastic residual $R_{t}$. The trend is used as a slowly varying macroscopic reference associated with thermodynamic-state evolution, whereas the residual preserves wear-related fluctuations that are more sensitive to incipient degradation. The overall architecture is shown in Figure 1.

As shown in Figure 1, the framework contains four sequential modules. Hybrid-Decomp separates $X_{t}$ into $T_{t}$ and $R_{t}$ while preserving structural transitions and suppressing spike-like artifacts. M-Linear then projects the trend into the future to obtain the deterministic baseline ${\hat{T}}_{t}$ with thermodynamic dependencies retained. Based on ${\hat{T}}_{t}$, ResDiff models the residual as a trend-conditioned stochastic process and generates a residual sample ensemble ${\tilde{R}}_{t}$, which provides residual-uncertainty context for downstream score-domain Dynamic Risk Band (DRB) construction. The GVAE module integrates ${\hat{T}}_{t}$ and ${\tilde{R}}_{t}$ for reconstruction-based anomaly scoring while learning latent sensor dependencies for diagnostic interpretation. In practical monitoring, these modules can be trained and calibrated offline, while online operation only requires window-wise trend prediction, residual risk updating, and anomaly scoring for incoming sensor segments.

Within this framework, the score-domain DRB provides a dynamic upper-bound reference for early warning based on the GVAE reconstruction-error trajectory, whereas the final anomaly decision is determined by the GVAE reconstruction error under a calibrated threshold. This separation allows stochastic residual variability to be modeled without directly destabilizing the point anomaly score. In addition, the learned sensor-dependency structure provides auxiliary maintenance-oriented diagnostic cues for sensor-level inspection.

### 2.2. Hybrid-Decomp: Gradient-Constrained Signal Decomposition

**Figure 2:**
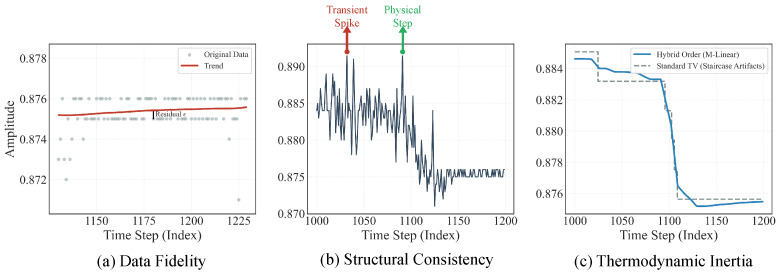
Mechanism of the Hybrid-Decomp module.

The decomposition of the raw condition-monitoring sequence $X_{t}$ is formulated as a structure-adaptive inverse problem. To separate the deterministic trend from complex background noise, Hybrid-Decomp introduces a gradient-consistency-constrained variational optimization process. Specifically, the macroscopic trend $T_{t}$ is estimated by solving the following hybrid-order trend-filtering objective:

(1) $$\begin{array}{cc} T_{t}=\arg\min_{T}( & \frac{1}{2}{∥X_{t}-T∥}_{2}^{2}\\ \; & +\lambda_{1}∥W_{cons}⊙\left(D_{1}T\right){∥}_{1}+\lambda_{2}{∥D_{2}T∥}_{1}) \end{array}$$

where $D_{1}$ and $D_{2}$ denote the first- and second-order difference matrices, respectively, $W_{cons}$ is an adaptive gradient-consistency matrix, ⊙ denotes the Hadamard product, and $\lambda_{1},\lambda_{2}$ are structural hyperparameters. As shown in Figure 2, the objective balances data fidelity, transition preservation, and structural smoothness.

The first term constrains the estimated trend to remain close to the raw observations, thereby preserving the dominant low-frequency operating-state trend reference. The second term introduces gradient consistency to distinguish sustained structural shifts from stochastic local fluctuations, allowing degradation-related step changes to be retained while suppressing spike-like artifacts. The third term imposes second-order sparsity to mitigate the staircase artifacts commonly induced by standard first-order trend filtering and to enforce piecewise-linear evolution consistent with thermodynamic inertia.

After optimization, the stochastic residual component is obtained as $R_{t}=X_{t}-T_{t}$, providing the decoupled high-frequency information for subsequent residual modeling and risk assessment.

### 2.3. M-Linear: Efficient Deterministic Trend Forecasting

**Figure 3:**
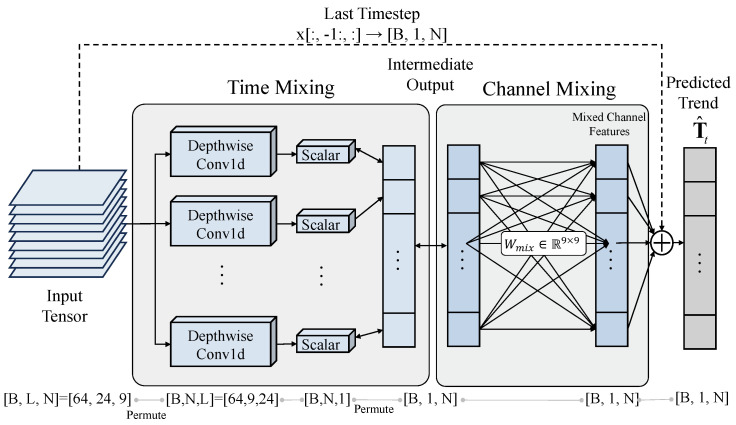
Structural details of the M-Linear framework.

To forecast the decoupled macroscopic trends accurately and efficiently, the proposed framework introduces the M-Linear (Multivariate Linear) architecture. As shown in Figure 3, M-Linear combines linear temporal projection with explicit multivariate coupling to forecast the decoupled macroscopic trend references. This behavior is consistent with the design goal of preserving multivariate thermodynamic evolution rather than relying on purely channel-independent trend extrapolation.

Let $T_{hist}\in R^{B\times L\times N}$ denote the input historical trend sequence derived from the Hybrid-Decomp module, where *B* is the batch size, *L* is the look-back window length, and *N* is the number of sensor nodes. The forecasting process begins with a temporal projection stage. A depthwise one-dimensional convolution (DW-Conv1d) with a kernel size of *L* independently maps the historical sequence of each channel into a scalar latent representation:

(2) $$H_{time}=\mathrm{DW}\text{-}\mathrm{Conv}1d\left(T_{hist}\right)\in R^{B\times 1\times N}$$

This stage captures the temporal inertia of each variable without introducing premature interchannel interference.

The latent features are then processed by a linear transformation across the channel dimension to model thermodynamic coupling:

(3) $$H_{coupled}=H_{time}W_{mix}^{⊤}+b_{channel}\in R^{B\times 1\times N}$$

where the dependency matrix $W_{mix}\in R^{N\times N}$ characterizes the interaction strength among sensor variables. In this way, M-Linear preserves the endogenous coupling structure that is neglected by channel-independent linear forecasting.

Finally, the future trend ${\hat{T}}_{t}$ is predicted through a residual connection anchored to the most recent historical observation $T_{hist,L}\in R^{B\times 1\times N}$:

(4) $${\hat{T}}_{t}=T_{hist,L}+H_{coupled}$$

This design improves forecasting continuity while providing a stable deterministic baseline for downstream residual generation and anomaly assessment.

### 2.4. ResDiff: Trend-Conditioned Stochastic Residual Generation

Microscopic tribological dynamics can introduce asymmetric, heteroscedastic, and heavy-tailed stochastic fluctuations into the residual domain. To characterize such residual uncertainty under the forecasted operating trend, we adopt ResDiff, a trend-conditioned generative framework based on Denoising Diffusion Probabilistic Models (DDPMs).

**Figure 4:**
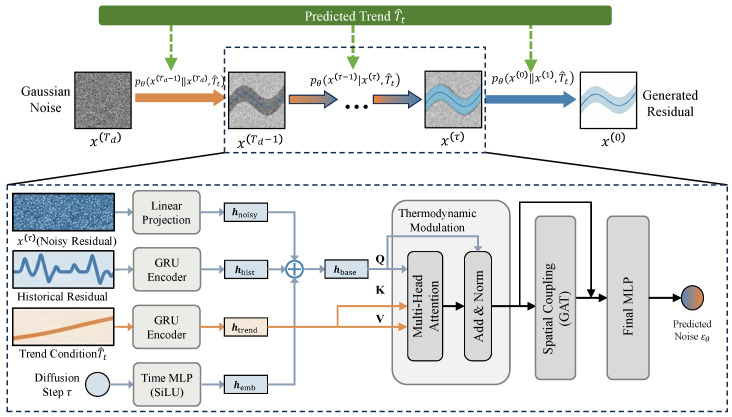
Architecture of the ResDiff module. The thick arrows in the upper panel indicate the reverse denoising trajectory, with the ellipsis representing intermediate reverse-diffusion steps; dashed green arrows indicate the predicted-trend conditioning input, and the blue and yellow solid paths in the lower module denote residual-state processing and trend-condition injection, respectively.

As shown in Figure 4, ResDiff learns the conditional distribution of residuals given the forecasted trend ${\hat{T}}_{t}$. At the diffusion level, the reverse process is conditioned on the trend to capture the state dependency of industrial residuals:

(5) $$\begin{array}{cc} p_{\theta } & \left(x^{\left(\tau -1\right)}|x^{\left(\tau \right)},{\hat{T}}_{t}\right)\\ \; & =N\left(x^{\left(\tau -1\right)};\mu_{\theta }\left(x^{\left(\tau \right)},\tau ,{\hat{T}}_{t}\right),\Sigma_{\tau }\right) \end{array}$$

where $x^{\left(\tau \right)}$ denotes the noisy residual state at diffusion step τ. By injecting ${\hat{T}}_{t}$ throughout the reverse trajectory, the model adapts residual generation to the instantaneous operating condition.

The conditional mean $\mu_{\theta }$ is parameterized by the proposed Graph-Evolved Denoising Network (GED-Net). Its Thermodynamic Modulation mechanism uses cross-attention to incorporate the trend embedding into residual denoising:

(6) $$\begin{array}{cc} \; & \mathrm{Attn}(h_{base},h_{trend})\\ \; & \;=\mathrm{Softmax}\left(\frac{\left(h_{base}W_{Q}\right){\left(h_{trend}W_{K}\right)}^{⊤}}{\sqrt{d_{k}}}\right)\left(h_{trend}W_{V}\right) \end{array}$$

where $h_{base}$ is derived from $x^{\left(\tau \right)}$ and the step embedding, and $h_{trend}$ denotes the trend embedding. This modulation allows the denoising process to vary with the macroscopic thermodynamic state.

To capture intersensor dependencies, the modulated features are further processed through a fully connected Graph Attention Network:

(7) $$h_{i}^{\prime }=\sigma \left(\sum_{j=1}^{N}a_{ij}W_{G}h_{j}\right)$$

where the attention coefficient $a_{ij}$ is computed as

(8) $$a_{ij}=\frac{\exp\left({\left(h_{i}W_{G}\right)}^{⊤}\left(h_{j}W_{G}\right)/\sqrt{d_{k}}\right)}{\sum_{l=1}^{N}\exp\left({\left(h_{i}W_{G}\right)}^{⊤}\left(h_{l}W_{G}\right)/\sqrt{d_{k}}\right)}$$

This fully connected formulation enables the model to learn latent coupling patterns directly from multivariate residual dynamics.

During inference, the trained model generates S=50 residual trajectories conditioned on the same trend ${\hat{T}}_{t}$, yielding the residual sample ensemble ${\tilde{R}}_{t}$. A residual uncertainty envelope is then obtained from this ensemble through quantile estimation, providing a state-dependent probabilistic context for subsequent anomaly assessment.

### 2.5. GVAE: Risk-Aware Anomaly Scoring with Spatial Dependency

**Figure 5:**
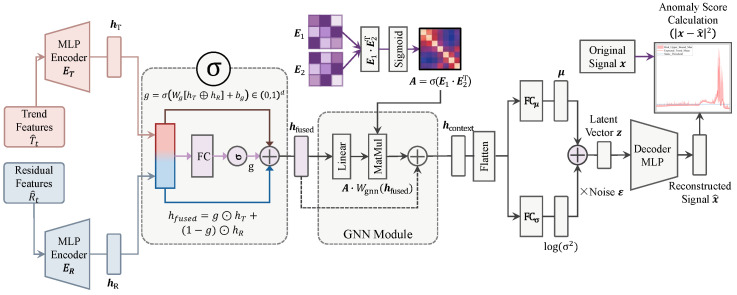
Schematic of the GVAE with graph learning.

To fuse deterministic operational states and stochastic perturbations for robust anomaly perception, the proposed dual-stream gated GVAE operates in the decoupled feature space derived from the preceding modules. As shown in Figure 5, directly concatenating these heterogeneous features would allow the high-amplitude trend component to dominate the representation. To address this mismatch, two independent multilayer perceptron (MLP) encoders are used to map the trend and residual inputs into separate latent embeddings $h_{T}$ and $h_{R}$.

Their integration is governed by an adaptive gating mechanism:

(9) $$g=\sigma \left(W_{g}\left[h_{T}⊕h_{R}\right]+b_{g}\right)$$

and the fused representation is obtained as follows:

(10) $$h_{fused}=g⊙h_{T}+\left(1-g\right)⊙h_{R}$$

This mechanism adaptively balances the contributions of the trend and residual streams under different operating conditions.

Based on the fused features, the GVAE learns a directed adjacency matrix A from data using trainable source and target embeddings $E_{1},E_{2}\in R^{N\times d}$:

(11) $$A=\sigma (E_{1}E_{2}^{⊤})$$

A graph neural network layer then processes $h_{fused}$ through graph message passing over the learned topology to capture intersensor dependency patterns. The network is optimized by minimizing the variational loss:

(12) $$L=E_{q(z|x)}\left[∥x-\hat{x}{∥}^{2}\right]+\beta_{KL}D_{KL}\left(q(z|x)∥p(z)\right)$$

where x corresponds to the normalized raw signal $X_{t}$, p(z) is the standard normal prior, and $\beta_{KL}$ controls the latent regularization strength.

During inference, the residual sample ensemble generated by ResDiff is passed through the GVAE scoring pathway to derive the score-domain Dynamic Risk Band (DRB) upper bound. After training, this inference process is implemented as a forward monitoring pass over each incoming sliding window, which is compatible with the 1 min sampling interval of the oil-monitoring system. For each of the *S* samples ${\tilde{R}}_{t}^{\left(s\right)}$, the trend–residual composite input is formed as ${\hat{X}}_{t}^{\left(s\right)}={\hat{T}}_{t}+{\tilde{R}}_{t}^{\left(s\right)}$. The trained GVAE reconstruction mapping D(·) then reconstructs this composite representation, and the score-domain DRB upper bound is defined as the *q*-th quantile of the resulting reconstruction-error distribution:

(13) $$\delta_{t}={\mathrm{Quantile}}_{q}\left({\left\{∥{\hat{X}}_{t}^{\left(s\right)}-D\left({\hat{T}}_{t},{\tilde{R}}_{t}^{\left(s\right)}\right){∥}^{2}\right\}}_{s=1}^{S}\right)$$

The observed deterministic reconstruction error is then evaluated against a calibrated threshold to produce the threshold-based anomaly decision, while the temporal evolution of the score-domain DRB upper bound $\delta_{t}$ provides an early-warning reference that complements this decision. When a system-level alarm is triggered, the learned adjacency matrix A is further used to provide sensor-level diagnostic cues by highlighting prominent residual dependencies among sensor channels.

## 3. Experiment

### 3.1. Dataset and Implementation Details

**Figure 6:**
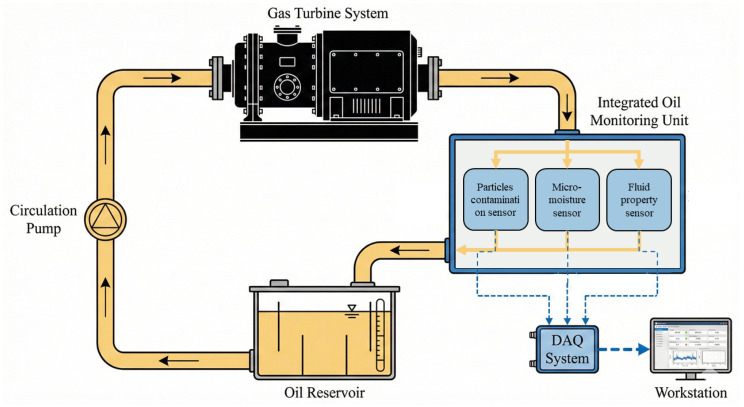
Experimental platform and multi-sensor layout.

The online monitoring data stream used in this study was acquired from the closed-loop lubrication system of an industrial gas turbine operating under extreme conditions. As shown in Figure 6, the integrated monitoring unit records a nine-dimensional signal vector, including four-scale particulate contamination (4 μm, 6 μm, 14 μm, and 21 μm), four thermodynamic properties (density, viscosity, dielectric constant, and temperature), and water content. The monitoring unit included an OPCom II particle contamination sensor (ARGO-HYTOS GmbH, Kraichtal-Menzingen, Germany), a LubCos H2Oplus II micro-moisture sensor (ARGO-HYTOS GmbH, Kraichtal-Menzingen, Germany), and an FPS2800B12C4 fluid property sensor (TE Connectivity Sensors/MEAS, Schaffhausen, Switzerland). All signals were synchronously sampled at a uniform interval of 1 min, covering a continuous oil-monitoring record from early operation to the built-in system-level alarm recorded in the dataset. Representative temporal sequences of the monitored lubricant data are provided in Supplementary Table S1.

Based on this continuous industrial monitoring record, a strict chronological protocol was adopted for model development and evaluation. For the M-Linear and ResDiff modules, the first four days of data were used for training to capture historical trends and operating variations. For the GVAE module, the reference data were further restricted to the earliest two days of the record to construct the unsupervised normal-pattern reference. This conservative choice avoids using later samples that may already contain degradation-related changes when learning the anomaly-scoring boundary. The subsequent 48 h of data were used as a unified test set for continuous condition monitoring and fault detection. For clarity in later evaluation and visualization, the time index of this two-day test interval was reinitialized from 1 to 2880, and the built-in system-level alarm timestamp occurred at index 1531.

The framework was implemented using a two-stage parameter-selection strategy that combines physics-aware manual initialization with Bayesian optimization for downstream learning modules. The computational experiments were implemented in Python 3.8 using PyTorch 1.13.1, with hyperparameter search performed using Optuna 4.5.0 and statistical baselines implemented using statsmodels 0.14.1. For Hybrid-Decomp, the structural hyperparameters were determined according to the physical characteristics of oil-monitoring signals, with stronger smoothness regularization assigned to viscosity to reflect its higher thermal inertia. For M-Linear, the look-back window and learning rate were selected using Optuna-based Bayesian search on the validation set. The generative and inferential modules were configured by balancing modeling capacity and computational efficiency, with ResDiff using 100 diffusion steps and GVAE adopting a latent dimension of 7. The test interval, built-in system-level alarm timestamp, and alarm-centered evaluation window were not used for model training or hyperparameter selection; these event-related annotations were used only for final evaluation and visualization. The complete hyperparameter settings for all modules are summarized in Supplementary Table S2.

### 3.2. Evaluation Metrics

The proposed framework was evaluated from three complementary perspectives: signal decoupling, trend forecasting, and anomaly detection. For trend forecasting, prediction accuracy was evaluated using root mean squared error (RMSE) and mean absolute error (MAE), which are standard measures of point-forecasting error [40]. For anomaly detection, true positive rate (TPR), false positive rate (FPR), and the F1-score were adopted to assess warning sensitivity, false-alarm behavior, and overall detection balance [41,42]. Computational efficiency was additionally evaluated using inference latency, parameter count, and floating-point operations (FLOPs). For the signal decoupling stage, the real-world industrial record does not provide independently measured trend components. Therefore, performance was assessed by jointly considering reconstruction fidelity, structural smoothness, and the mitigation of staircase artifacts. In particular, structural smoothness was quantified by second-order total variation (TV2):

(14) $$\mathrm{Smoothness}=\frac{1}{T_{total}}\sum_{t=2}^{T_{total}-1}∥T_{t+1}-2T_{t}+T_{t-1}∥$$

where $T_{total}$ denotes the sequence length and $T_{t}$ is the estimated trend at time step *t*. This metric favors piecewise-linear evolution consistent with the physical inertia of fluid parameters.

Comparative experiments were conducted against representative baselines at the three corresponding stages. For signal decomposition, Hybrid-Decomp was compared with STL [10], VMD [11], and standard $\ell_{1}$ trend filtering [12]. For anomaly detection, ResAD-Net was benchmarked against LSTM-VAE [43], OmniAnomaly [44], USAD [45], GDN [33], MTAD–GAT [34], and a forecasting-based PatchTST variant [46]. For deterministic trend forecasting, M-Linear was evaluated against ARIMA [18], DLinear [17], LSTM [19], PatchTST [46], and iTransformer [16]. To ensure fair comparisons, all baseline models were implemented using their recommended settings, while the look-back window (L=24) and prediction horizon (H=1) were standardized across the deep-learning models.

## 4. Results and Discussion

### 4.1. Performance Evaluation of Signal Decoupling

To evaluate whether the proposed decomposition can provide a reliable input for downstream forecasting and anomaly detection, Hybrid-Decomp was compared with STL, VMD, and standard $\ell_{1}$ trend filtering. As summarized in Table 1, the proposed method achieves the best overall balance among reconstruction fidelity, structural smoothness, and correlation with the original signals. In particular, it yields the lowest average mean squared error (MSE) and the highest average correlation, while maintaining a low smoothness value, indicating that the extracted trends remain both faithful to the raw observations and structurally regular.

**Figure 7:**
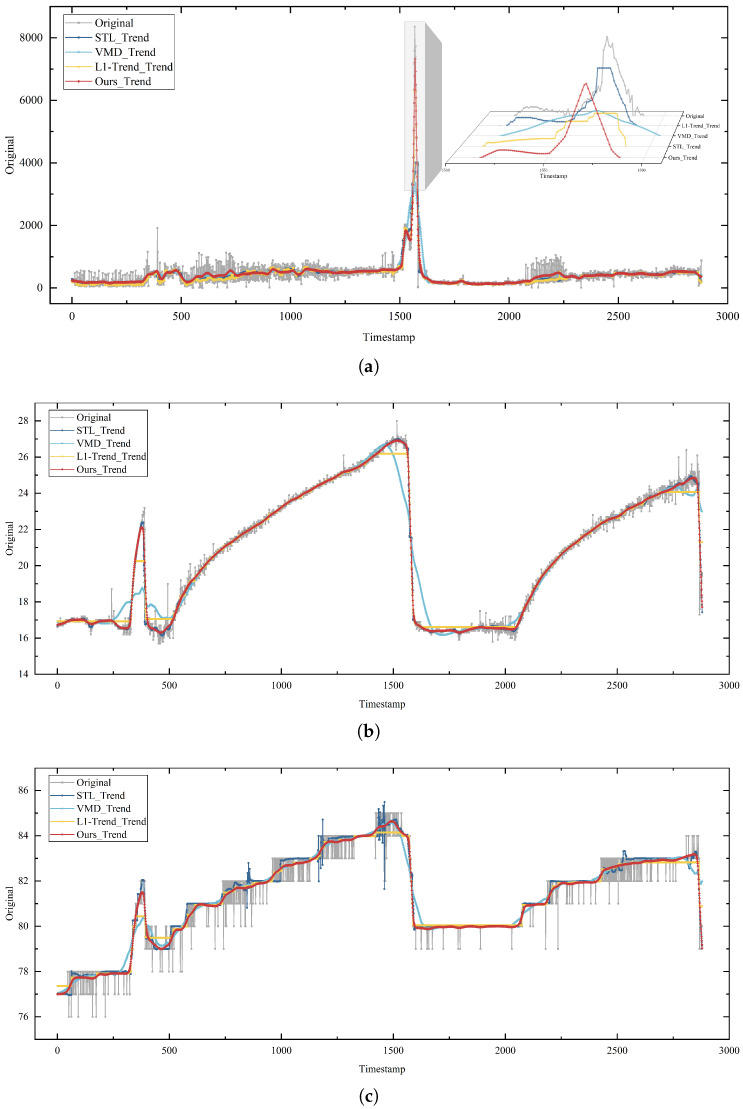
Comparative signal-decomposition results for representative channels: (**a**) 4 μm particle concentration; (**b**) viscosity; and (**c**) water content.

The representative decomposition results in Figure 7 further illustrate the different failure modes of the baselines. In the 4 μm channel, the enlarged view around the abrupt surge shows that the proposed method follows the local abrupt increase more closely, whereas the competing methods either smooth the transition excessively or fail to track the local structure accurately. In the viscosity channel, VMD exhibits a visible deviation from the underlying macroscopic evolution, while standard $\ell_{1}$ trend filtering produces staircase-like plateaus that are inconsistent with the continuous thermodynamic behavior of the variable. In the moisture channel, STL introduces unnatural local jumps at several positions. By contrast, the proposed method preserves the dominant trend while avoiding these distortions. Additional decomposition results and quantitative metrics for the remaining sensor channels are provided in Section S3 of the Supplementary Materials (Supplementary Table S3 and Supplementary Figure S1).

To further examine the decomposition behavior under a setting where the underlying trend is known, a controlled synthetic validation was conducted with three representative operating patterns: slow drift, abrupt degradation-like transition, and recovery-like trend reversal. Each signal was generated by combining a known macroscopic trend with correlated heavy-tailed residual fluctuations and sparse impulses, and the estimated trend was compared directly with the known trend. As summarized in Supplementary Table S4 and Supplementary Figure S2, the proposed method achieved the best overall average trend normalized root mean squared error (nRMSE) (0.0416 ± 0.0113), transition nRMSE (0.0482 ± 0.0110), trend correlation (0.9926 ± 0.0042), and residual leakage (14.43 ± 3.86%) across the controlled cases. These complementary results support the use of Hybrid-Decomp as a macroscopic trend-reference extractor before residual-domain modeling.

### 4.2. Evaluation of Trend Forecasting Capabilities

**Figure 8:**
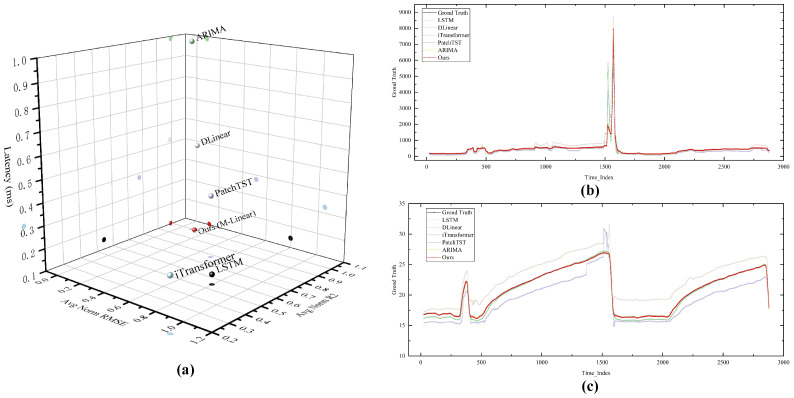
Efficiency–accuracy trade-off and representative forecasting trajectories: (**a**) aggregate accuracy–latency comparison, (**b**) 4 μm forecasting trajectory, and (**c**) viscosity forecasting trajectory. In panel (**a**), each colored dot denotes an individual forecasting model.

The forecasting performance of M-Linear was evaluated against statistical, linear, recurrent, and transformer-based baselines. As summarized in Table 2 and Figure 8a, ARIMA achieves the lowest MAE and RMSE and the highest $R^{2}$. M-Linear remains close in coefficient of determination ($R^{2}=0.992$ versus 0.994 for ARIMA, an absolute difference of 0.002) and reduces the average inference latency from 0.990 ms to 0.103 ms, while retaining an explicit multivariate coupling structure. Compared with DLinear, the additional complexity mainly comes from the lightweight channel-mixing step used to represent intersensor trend coupling; the resulting parameter count and FLOPs remain within the lightweight linear-model range.

The representative trajectories in Figure 8b,c further show differences that are not fully reflected by the aggregate metrics. In the 4 μm channel, LSTM, iTransformer, and PatchTST exhibit clear overestimation around the small pre-peak fluctuation, producing an unrealistic double-peak pattern before the main surge. ARIMA and DLinear follow the overall trend more closely but still show slight overestimation near the dominant peak. By comparison, M-Linear provides a more stable response to both the local fluctuation and the main pronounced rise. In the viscosity channel, the recurrent and transformer-based baselines show more visible trajectory deviations, whereas M-Linear remains closer to the reference macroscopic trend trajectory. Additional forecasting results for the remaining sensor channels are provided in Section S5 of the Supplementary Materials (Supplementary Table S5 and Supplementary Figure S3).

### 4.3. Dual-Dimension Diagnostic Evaluation: Early Warning and Sensor-Level Diagnostic Association

#### 4.3.1. Residual Distribution Diagnostics and Uncertainty Envelopes

**Figure 9:**
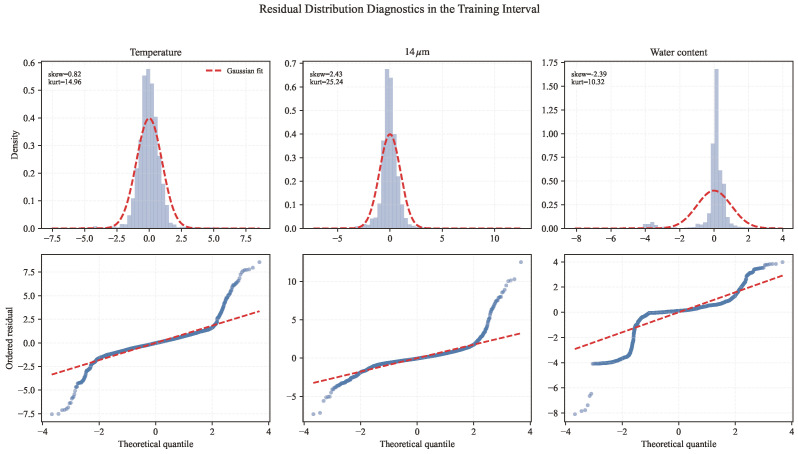
Residual distribution diagnostics for representative monitored parameters in the training interval. The upper panels compare empirical residual histograms with Gaussian fits, and the lower panels show Q-Q plots against a Gaussian reference. Blue bars/points represent empirical residual samples, whereas red dashed curves/lines denote Gaussian fits or Gaussian-reference lines.

Before interpreting the residual uncertainty envelopes, the residual distributions obtained after trend–residual decoupling were examined in the training interval. Figure 9 presents representative residual diagnostics for temperature, 14 μm particle concentration, and water content, covering thermodynamic, particulate, and fluid-related monitoring channels.

The representative residuals show clear departures from a symmetric Gaussian pattern. Their skewness values are 0.82, 2.43, and −2.39, and their excess kurtosis values are 14.96, 25.24, and 10.32, respectively; the Jarque–Bera tests reject normality for all three channels (p<0.001). The complete sensor-wise statistics are summarized in Supplementary Table S6, and extended training-interval residual diagnostics for the remaining sensors are provided in Supplementary Figure S4. The test-time evolution of residual volatility and skewness across all sensors is further provided in Supplementary Figure S5. Together, these results indicate that the residual domain is non-Gaussian and time-varying, supporting the need for a state-adaptive probabilistic residual model.

**Figure 10:**
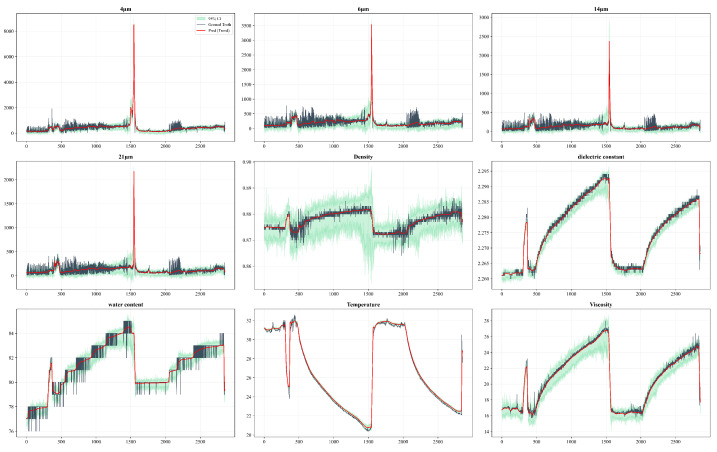
Visualization of residual-level trend-conditioned uncertainty envelopes across monitored parameters.

Motivated by these residual diagnostics, the ResDiff module generates trend-conditioned residual uncertainty envelopes as sensor-level probabilistic references for interpreting residual departures. As shown in Figure 10, the resulting envelope exhibits two salient characteristics beyond conventional static thresholds or symmetric Gaussian intervals, providing an uncertainty-aware context for interpreting residual departures under changing operating states.

Consistent with the residual diagnostics, the residual uncertainty envelope does not impose a fixed symmetric interval. Conditioned on the forecasted thermodynamic state, the envelope contracts during relatively stable operating periods and expands around transient fluctuations, thereby adapting to the time-varying stochasticity of the residual domain. In the particulate concentration channels, the upper boundary also extends more prominently than the lower boundary, reflecting the one-sided heavy-tailed behavior associated with wear-related particle growth.

#### 4.3.2. Quantitative Performance Assessment and Risk-Aware Scoring

To evaluate temporal warning behavior under industrial conditions, the final 48 h of the dataset were used as a chronological test set. Apart from the built-in system-level alarm event at index 1531, this record does not contain pointwise fault labels. Therefore, an alarm-referenced evaluation assumption was adopted, in which the initial 1440 time steps were used as the pre-alarm reference interval for false-positive evaluation, and the window [1520, 1580], where pronounced multi-sensor variations occur around the alarm trigger, was used as the alarm-centered evaluation window. The main ResAD-Net detection results are reported using repeated-run mean performance, whereas the temporal trajectories are shown for a representative run close to this mean performance.

Table 3 summarizes the quantitative comparison between the proposed trend-referenced anomaly score and the raw signal reconstruction baseline under the same chronological evaluation protocol. Across repeated runs, both the proposed metric and the raw signal reconstruction baseline showed no observed false positives within the predefined pre-alarm reference interval. Meanwhile, the proposed metric achieved a mean true-positive rate of 97.00% over the alarm-centered evaluation window, consistent with the alarm event recorded in the industrial data.

**Figure 11:**
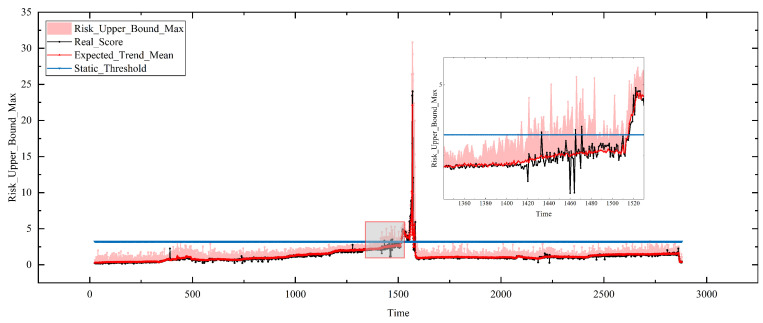
Temporal evolution of the anomaly score and score-domain Dynamic Risk Band upper bound.

The temporal evolution of the anomaly score and the score-domain Dynamic Risk Band upper bound are shown in Figure 11. During the early pre-alarm reference stage, transient enlargements of the DRB absorb localized environmental fluctuations without triggering false alarms. A different pattern emerges near the alarm-centered evaluation window, where the DRB upper bound expands and the calibrated anomaly score increases persistently. The threshold-based anomaly decision is triggered when the calibrated anomaly score crosses the calibrated anomaly threshold at index 1516, 15 min before the built-in system-level alarm timestamp at index 1531.

#### 4.3.3. Residual-Domain Diagnostic Association via Graph Learning

**Figure 12:**
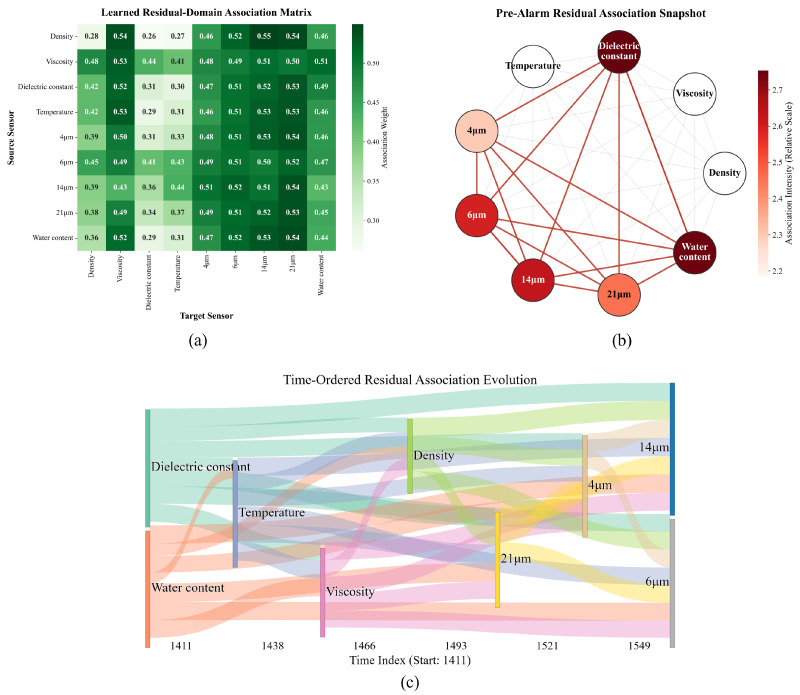
Residual-domain diagnostic association: (**a**) learned dependency matrix; (**b**) pre-alarm dependency snapshot; (**c**) temporal evolution of dependency patterns.

The association-based diagnostic capability of the framework is realized through the GVAE graph structure learner, which operates on the decoupled residual representations. As shown in Figure 12a, the learned dependency matrix summarizes residual-domain associations among sensor channels and provides sensor-level cues for diagnostic inspection.

The pre-alarm dependency snapshot in Figure 12b further highlights a diagnostic association involving water content, dielectric constant, and particulate channels. Around index 1411, the residual behavior of water content and dielectric constant begins to show a sustained shift, and the learned dependency pattern is consistent with the observed oil-monitoring changes around the system-level alarm. The temporal evolution of this dependency pattern is summarized in Figure 12c. This association serves as a maintenance cue for prioritizing sensor-level inspection, while the quantitative detection metrics in Table 3 are obtained from the thresholded anomaly-score sequence, whose first threshold crossing occurs at index 1516.

### 4.4. Comparative Analysis of Early Fault Warning

To evaluate ResAD-Net under nonstationary and multivariate coupled oil-monitoring conditions, comparative experiments were conducted against six representative anomaly-detection baselines in the early-warning task under the same chronological evaluation protocol.

As reported in Table 4, the proposed framework achieves the best overall balance between sensitivity and specificity, with a mean F1-score of 0.985, a mean TPR of 97.00%, and no observed false positives in the predefined pre-alarm reference interval. In contrast, the forecasting-based PatchTST baseline suffers from excessive false alarms, while OmniAnomaly, LSTM-VAE, and USAD show severe under-detection. The graph-based baselines GDN and MTAD-GAT perform better than the reconstruction-based methods but remain clearly below the proposed framework in overall anomaly-identification capability.

**Figure 13:**
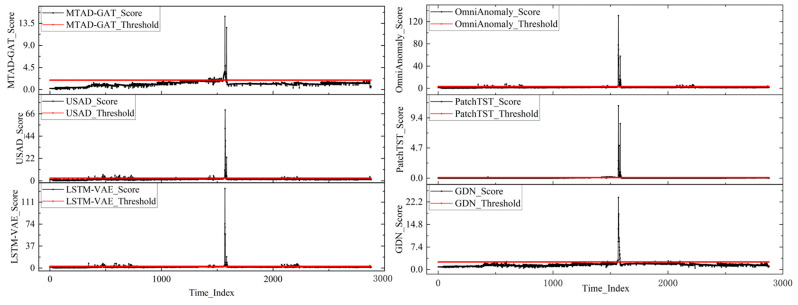
Representative anomaly-score trajectories of baseline models under the same chronological evaluation protocol.

The anomaly-score trajectories in Figure 13 further illustrate these differences. PatchTST frequently responds to pre-alarm fluctuations, resulting in a rigid thresholding pattern with limited robustness to nonstationary noise. The probabilistic and reconstruction-based baselines show delayed or weakened responses to the pre-alarm evolution stage.

### 4.5. Ablation Studies

An ablation study was conducted to assess the contributions of the core functional designs in ResAD-Net. To preserve the input–output consistency of the dual-stream framework, the ablation variants were implemented as replacement-based comparisons rather than direct module deletion. Specifically, w/o Decomp replaces the trend–residual decoupling with raw-signal input, w/o Diffusion replaces ResDiff with Gaussian residual sampling, and w/o Graph keeps the dual-stream variational-autoencoder backbone while removing graph learning.

As shown in Table 5, each replacement variant produces a substantial reduction in mean detection performance, indicating that trend–residual decoupling, probabilistic residual modeling, and dependency learning each contribute to the overall warning capability of the framework. In particular, replacing ResDiff with Gaussian residual sampling reduces the mean F1-score from 0.985 to 0.394, corresponding to a relative F1 drop of 60.00%, which is consistent with the skewed, heavy-tailed, and time-varying residual behavior observed in Figure 9, Supplementary Table S6, and Supplementary Figures S4 and S5.

**Figure 14:**
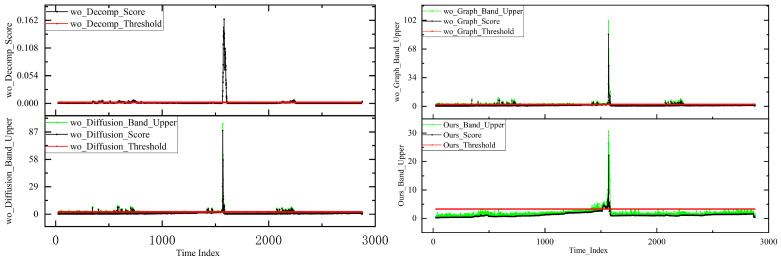
Representative anomaly-score trajectories of ablation variants: raw-signal replacement, Gaussian residual replacement, graph-learning removal, and full ResAD-Net.

The anomaly-score trajectories in Figure 14 further clarify the role of each functional design. Under raw-signal replacement, the model becomes more vulnerable to macroscopic operational variations. Under Gaussian residual replacement, the framework loses the state-adaptive residual uncertainty context generated by ResDiff for downstream score-domain DRB construction and becomes less effective in characterizing nonstationary residual changes. Without graph learning, the model processes sensor responses in a more isolated manner, which weakens system-level anomaly perception.

From a deployment perspective, the proposed framework is intended for minute-level condition monitoring rather than high-frequency control. The main model training and hyperparameter selection can be performed offline, while online monitoring only requires periodic forward inference. In the present nine-sensor setting, the multivariate channel-mixing and residual-domain dependency operations remain small-scale; larger sensor networks would require additional pipeline-level latency and memory profiling.

## 5. Conclusions

In real-world industrial condition monitoring, multi-sensor lubricating oil data often exhibit pronounced nonstationary thermodynamic drifts, which can obscure the weak residual fluctuations associated with incipient degradation. To address this challenge, the proposed ResAD-Net framework adopts a trend–residual decoupling strategy and performs residual-centered modeling for early fault warning. By separating deterministic macroscopic trend references from stochastic wear-related residuals, the framework provides a more suitable representation for capturing weak early-stage abnormality signatures in coupled monitoring signals.

The experimental results demonstrate the effectiveness of the proposed framework in both temporal warning and diagnostic interpretation. In temporal warning, trend-conditioned residual modeling and the score-domain Dynamic Risk Band enable the system to detect residual distributional changes before clear macroscopic deviations emerge, achieving an average F1-score of 0.985 and a mean TPR of 97.00%, with no observed false positives in the predefined pre-alarm reference interval of this finite test set. From a diagnostic perspective, the learned residual-domain dependency structure provides diagnostic association cues for interpreting oil-monitoring changes around the system-level alarm, highlighting associations among early water-related changes and particulate-channel responses in a manner consistent with the lubrication-related alarm context. The learned graph therefore serves as an association-based maintenance cue for sensor-level inspection.

Overall, the results indicate that residual-centered learning provides a practical route for interpreting nonstationary multi-sensor monitoring streams in early fault warning and health management. The historical record analyzed in this study uses the system-level alarm timestamp as the external event marker, and broader validation requires datasets with independently verified nominal and anomalous periods. Future work will therefore focus on extending this residual-driven monitoring paradigm to additional equipment types and operating regimes under such verified evaluation settings.

## Figures and Tables

**Table 1:** Quantitative performance comparison of signal-decomposition methods.

Model	Avg. MSE	Avg. Smoothness	Avg. Correlation
Ours	$1.17\times 10^{-1}$	$1.29\times 10^{-3}$	0.938
L1-Trend	$1.27\times 10^{-1}$	$2.34\times 10^{-2}$	0.9345
VMD	$2.48\times 10^{-1}$	$1.75\times 10^{-3}$	0.8625
STL	$2.59\times 10^{-1}$	$5.12\times 10^{-3}$	0.8479

**Table 2:** Statistical summary of average trend forecasting performance.

Model	MAE	RMSE	$R^{2}$	Latency (ms)	Params (M)	FLOPs (G)
Ours (M-Linear)	$3.06\times 10^{-2}$	$1.16\times 10^{-1}$	0.992	0.103	$3.15\times 10^{-4}$	$9.35\times 10^{-5}$
ARIMA	$5.25\times 10^{-3}$	$8.57\times 10^{-2}$	0.994	0.990	-	-
DLinear	$4.30\times 10^{-2}$	$1.42\times 10^{-1}$	0.988	0.517	$2.25\times 10^{-4}$	$4.32\times 10^{-7}$
PatchTST	$1.65\times 10^{-1}$	$5.11\times 10^{-1}$	0.776	0.340	$1.38\times 10^{-1}$	$1.20\times 10^{-3}$
iTransformer	$7.44\times 10^{-1}$	1.05	0.094	0.327	$6.86\times 10^{-2}$	$1.96\times 10^{-3}$
LSTM	$6.51\times 10^{-1}$	$7.96\times 10^{-1}$	0.543	0.144	$1.98\times 10^{-2}$	$9.00\times 10^{-4}$

**Table 3:** Quantitative evaluation of anomaly detection scoring protocols.

Metric Source	TPR (%)	FPR (%)	F1-Score
ResAD-Net (Proposed)	97.00±1.39	0.00±0.00	0.985±0.007
Raw Score	91.00±1.90	0.10±0.08	0.941±0.019

**Table 4:** Quantitative performance comparison of anomaly-detection models.

Model	TPR	FPR	F1-Score
ResAD-Net	97.00%	0.00%	0.985
OmniAnomaly	23.30%	2.50%	0.254
PatchTST	100%	11.04%	0.43
LSTM-VAE	36.70%	4.72%	0.293
USAD	33.30%	4.44%	0.278
GDN	41.70%	0.28%	0.562
MTAD-GAT	61.70%	0.69%	0.692

**Table 5:** Comparison of anomaly-detection performance across ablation variants, with F1 drops reported relative to the full ResAD-Net model; downward arrows indicate the relative decrease in F1-score.

Model	TPR (%)	FPR (%)	F1-Score	Relative F1 Drop
Proposed	97.00±1.39	0.00±0.00	0.985±0.007	-
w/o Decomp	68.33±1.18	4.65±0.11	0.488±0.007	↓ 50.46%
w/o Diffusion	51.67±2.64	4.63±0.21	0.394±0.024	↓ 60.00%
w/o Graph	21.67±2.64	11.04±0.35	0.112±0.015	↓ 88.63%

## Data Availability

The data that support the findings of this study are not publicly available due to confidentiality restrictions.

## Supplementary Materials

The following supporting information can be downloaded at https://www.mdpi.com/article/10.3390/s26123779/s1. Table S1: Representative sequences of the monitored lubricant data over time; Table S2: Unified hyperparameter settings for the proposed ResAD-Net framework; Table S3: Detailed decomposition metrics for individual sensors; Table S4: Controlled synthetic decomposition validation; Table S5: Detailed trend forecasting performance metrics for individual sensors; Table S6: Residual distribution statistics for individual sensors; Figure S1: Extended decomposition results; Figure S2: Controlled synthetic decomposition validation; Figure S3: Extended trend forecasting results; Figure S4: Extended residual distribution diagnostics; Figure S5: Test-time residual volatility and skewness diagnostics.

## Abbreviations

The following abbreviations are used in this manuscript:

DDPM	Denoising Diffusion Probabilistic Model
DRB	Dynamic Risk Band
GAT	Graph Attention Network
GED-Net	Graph-Evolved Denoising Network
GVAE	Graph Variational Autoencoder
PHM	Prognostics and Health Management
