# Supplementary material for: Trend-Conditioned Residual Learning for Early Fault Warning in Nonstationary Multi-Sensor Oil Monitoring
Source: Sensors (Basel). 2026 Jun 13;26(12):3779. doi: 10.3390/s26123779 (PMC13307168; doi:10.3390/s26123779)
Supplement: Supplementary file 1 [file sensors-26-03779-s001.zip › sensors-4329394-supplementary.pdf]

Supplementary Materials for "Trend-Conditioned Residual Learning for Early Fault Warning in Nonstationary Multi-Sensor Oil Monitoring"

Huaqing Li, Yongxu Chen, Yitian Wang, Changlin Wu*

* Corresponding author. Email: wuchanglin@hnnu.edu.cn

S1 Detailed Characteristics of the Industrial Dataset

As a supplement to Section 3.1 of the main manuscript, this section provides detailed temporal sequences of the monitored lubricant data.

Table S1: Representative sequences of the monitored lubricant data over time.

**Time**	**Density** (g/cm^3)	**Viscosity** (cSt)	**Dielectric Constant**	**Temperature** (°C)	**4μm** (pcs/mL)	**6μm** (pcs/mL)	**14μm** (pcs/mL)	**21μm** (pcs/mL)	**Moisture (Water Content)** (mg/kg)
00:00:14	0.878	28.5	2.284	19.9	555	227	149	134	79
00:01:14	0.880	28.2	2.285	19.9	457	167	111	100	79
00:02:14	0.887	27.6	2.284	19.9	584	256	177	159	79
00:03:14	0.887	27.6	2.284	19.9	581	253	163	142	79
⋮	⋮	⋮	⋮	⋮	⋮	⋮	⋮	⋮	⋮
23:58:14	0.889	29.0	2.286	18.9	386	132	84	74	79
23:59:14	0.888	28.9	2.286	18.9	641	315	208	174	79

S2 Experimental Setup and Hyperparameter Configurations

To support reproducibility of the proposed ResAD-Net framework, the complete hyperparameter configurations for each analytical module are listed below.

Table S2: Unified hyperparameter settings for the proposed ResAD-Net framework.

Module	Parameter	Value
**Hybrid-Decomp**	Sparsity Weight (λ_1)	3.0
	Smoothness Weight (λ_2)	5.0 / 100.0
	Consistency Threshold (α)	3.0
**M-Linear**	Input Sequence Length (L)	24
	Prediction Horizon (H)	1
	Learning Rate	0.00675
	Batch Size	64
	Weight Decay	1.90×10^{-7}
**ResDiff**	Hidden Dimension	128
	Diffusion Steps (T_d)	100
	Noise Schedule Start	0.0001
	Noise Schedule End	0.05
	GNN Heads	4
**GVAE**	Latent Dimension (d_z)	7
	Risk Quantile (q)	95
	Warm-up Cut	24

S3 Extended Evaluation of Signal Decoupling (Hybrid-Decomp)

Complementing the selected visualizations in Section 4.1 of the main text, which highlight 4μm, viscosity, and Moisture (Water Content), Figure S1 presents the comprehensive signal-decomposition results across the remaining six monitoring variables. Table S3 further provides the detailed quantitative evaluation metrics for the decomposition stage.

Table S3: Detailed decomposition metrics for individual sensors; MSE denotes mean squared error. The best results are **bolded**, and the second-best results are <u>underlined</u>.

Sensor	Model	MSE	Smoothness	R^2
Density	STL	<u>1.46×10^{-6}</u>	<u>6.32×10^{-6}</u>	<u>0.934</u>
	VMD	1.47×10^{-6}	6.97×10^{-6}	**0.936**
	L1-Trend	1.54×10^{-6}	2.03×10^{-5}	0.932
	**Ours**	$\mathbf{1.43 \times 10^{-6}}$	$\mathbf{2.40 \times 10^{-6}}$	<u>0.934</u>
Viscosity	STL	<u>1.14×10^{-1}</u>	8.90×10^{-3}	<u>0.995</u>
	VMD	6.85×10^{-1}	$\mathbf{1.58 \times 10^{-3}}$	0.971
	L1-Trend	1.68×10^{-1}	1.83×10^{-2}	0.994
	**Ours**	$\mathbf{8.42 \times 10^{-2}}$	<u>2.76×10^{-3}</u>	**0.997**
Dielectric	STL	<u>8.52×10^{-7}</u>	8.93×10^{-6}	**0.996**
	VMD	4.83×10^{-6}	$\mathbf{5.41 \times 10^{-6}}$	0.977
	L1-Trend	1.49×10^{-6}	6.06×10^{-5}	<u>0.994</u>
	**Ours**	$\mathbf{7.76 \times 10^{-7}}$	<u>7.77×10^{-6}</u>	**0.996**
Temperature	STL	2.81×10^{-1}	7.87×10^{-3}	0.991
	VMD	1.38×10^{0}	$\mathbf{6.73 \times 10^{-4}}$	0.954
	L1-Trend	<u>1.77×10^{-1}</u>	1.80×10^{-2}	<u>0.995</u>

Continued on next page

Table S3: Detailed decomposition metrics for individual sensors (Continued).

**Sensor**	**Model**	**MSE**	**Smoothness**	R^2
	**Ours**	$\mathbf{7.10 \times 10^{-2}}$	3.17×10^{-3}	**0.998**
4μm	STL	7.02×10^{4}	3.62	0.867
	VMD	8.92×10^{4}	0.940	0.826
	L1-Trend	2.49×10^{4}	15.5	0.956
	**Ours**	$\mathbf{2.35 \times 10^{4}}$	**0.852**	**0.957**
6μm	STL	2.06×10^{4}	0.959	0.729
	VMD	2.01×10^{4}	0.630	0.729
	L1-Trend	1.14×10^{4}	9.85	0.859
	**Ours**	$\mathbf{1.04 \times 10^{4}}$	**0.387**	**0.868**
14μm	STL	1.34×10^{4}	0.331	0.512
	VMD	9.30×10^{3}	0.438	0.698
	L1-Trend	5.17×10^{3}	6.87	0.846
	**Ours**	$\mathbf{4.93 \times 10^{3}}$	**0.285**	**0.851**
21μm	STL	8.83×10^{3}	0.638	0.628
	VMD	7.41×10^{3}	0.381	0.702
	L1-Trend	3.85×10^{3}	6.35	0.858
	**Ours**	$\mathbf{3.70 \times 10^{3}}$	**0.266**	**0.862**
Water Content	STL	1.61×10^{-1}	3.79×10^{-2}	0.979
	VMD	2.30×10^{-1}	2.35×10^{-3}	0.970
	L1-Trend	1.67×10^{-1}	1.22×10^{-2}	0.979
	**Ours**	$\mathbf{1.47 \times 10^{-1}}$	$\mathbf{1.26 \times 10^{-3}}$	**0.981**

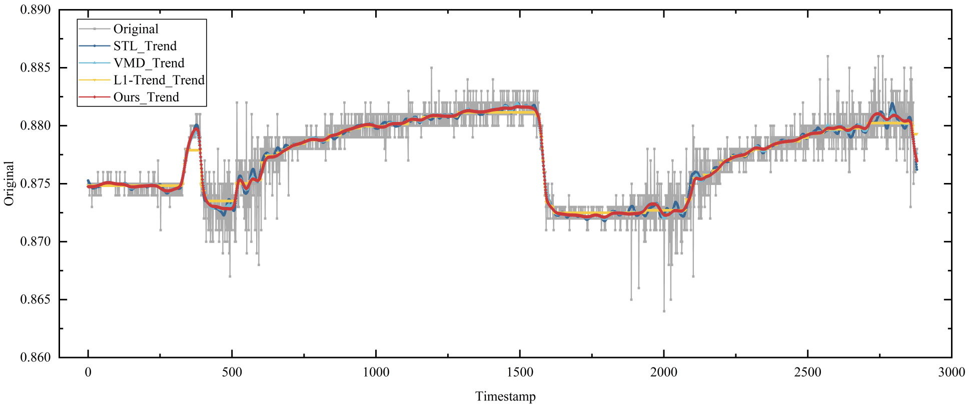

(a) Density

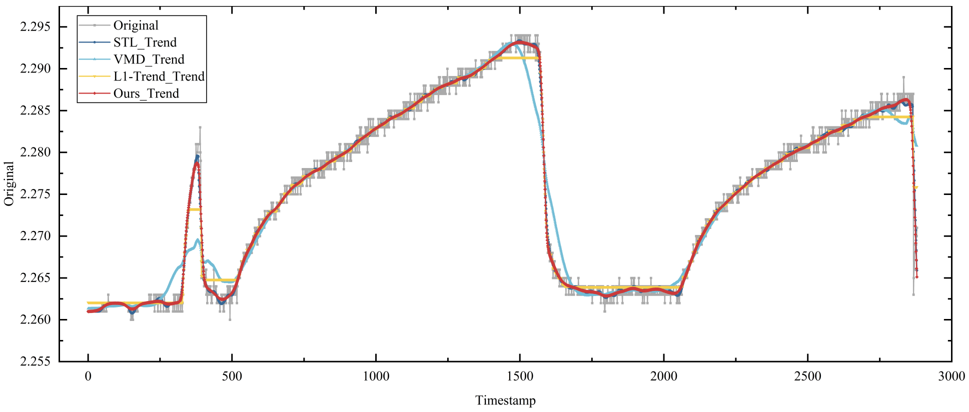

(b) Dielectric Constant

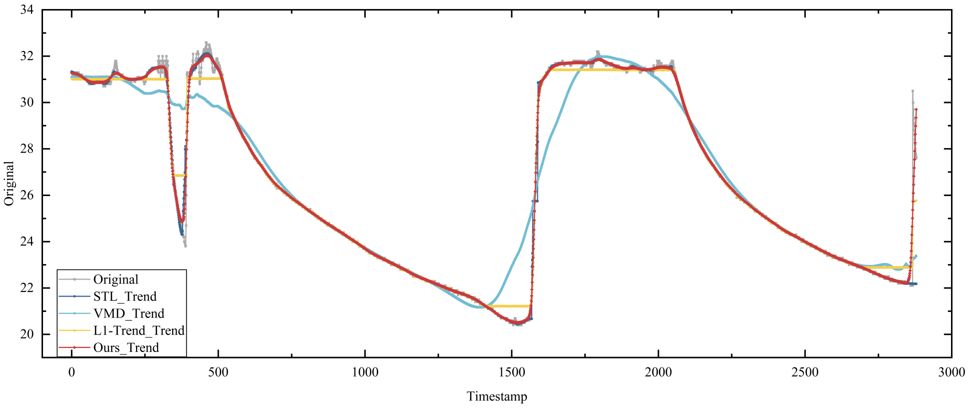

(c) Temperature

Figure S1: Comparative results of signal-decomposition methods on extended monitoring parameters.

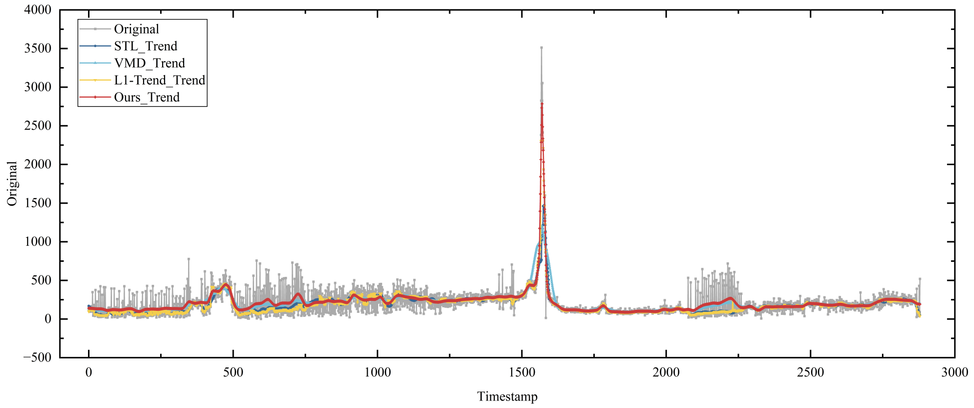

(d) 6μm

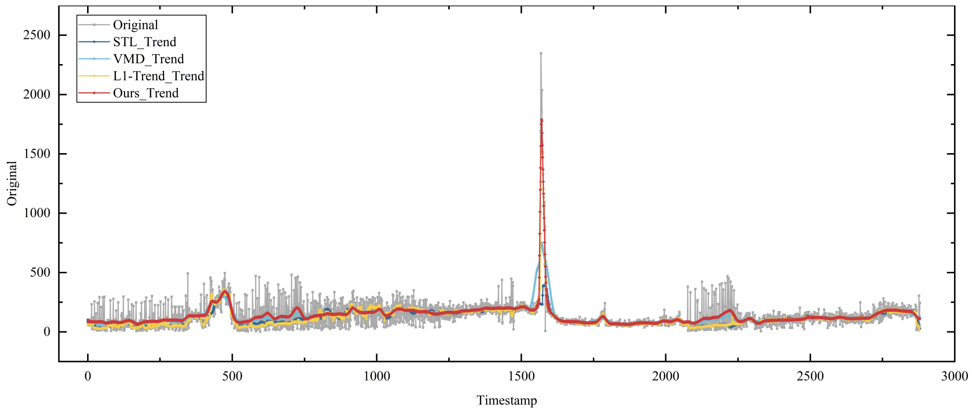

(e) 14μm

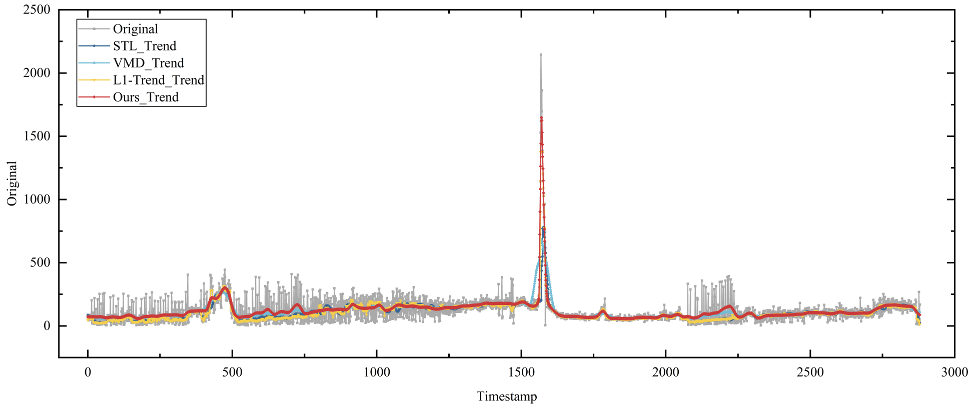

(f) 21μm

Figure S1: Comparative results of signal-decomposition methods on extended monitoring parameters (cont.).

S4 Controlled Synthetic Decomposition Validation

To complement the decomposition evaluation on the industrial monitoring record, a controlled synthetic study was conducted in which the macroscopic trend and residual component were known by construction. Three representative scenarios were considered: slow drift, abrupt degradation-like transition, and recovery-like trend reversal. The observed signal was generated by adding correlated heavy-tailed residual fluctuations and sparse impulses to the known trend. The compared methods included STL, VMD, ℓ_1 trend filtering, and the proposed Hybrid-Decomp. Performance was evaluated using trend normalized root mean squared error (nRMSE), transition-region nRMSE, trend correlation, and residual leakage, where residual leakage measures the variance of the trend-estimation error relative to the variance of the known residual component.

Table S4: Controlled synthetic decomposition validation with known trend–residual construction. Values are reported as mean ± std over twelve trials from three scenarios.

**Method**	**Trend nRMSE**	**Transition nRMSE**	**Trend Correlation**	**Residual Leakage (%)**
STL	0.0526 ± 0.0134	0.0530 ± 0.0162	0.9876 ± 0.0070	23.59 ± 6.38
VMD	0.0542 ± 0.0236	0.0750 ± 0.0406	0.9881 ± 0.0105	30.28 ± 26.06
L1-Trend	0.0589 ± 0.0131	0.0664 ± 0.0164	0.9896 ± 0.0052	31.17 ± 10.36
**Proposed**	$\mathbf{0.0416 \pm 0.0113}$	$\mathbf{0.0482 \pm 0.0110}$	$\mathbf{0.9926 \pm 0.0042}$	$\mathbf{14.43 \pm 3.86}$

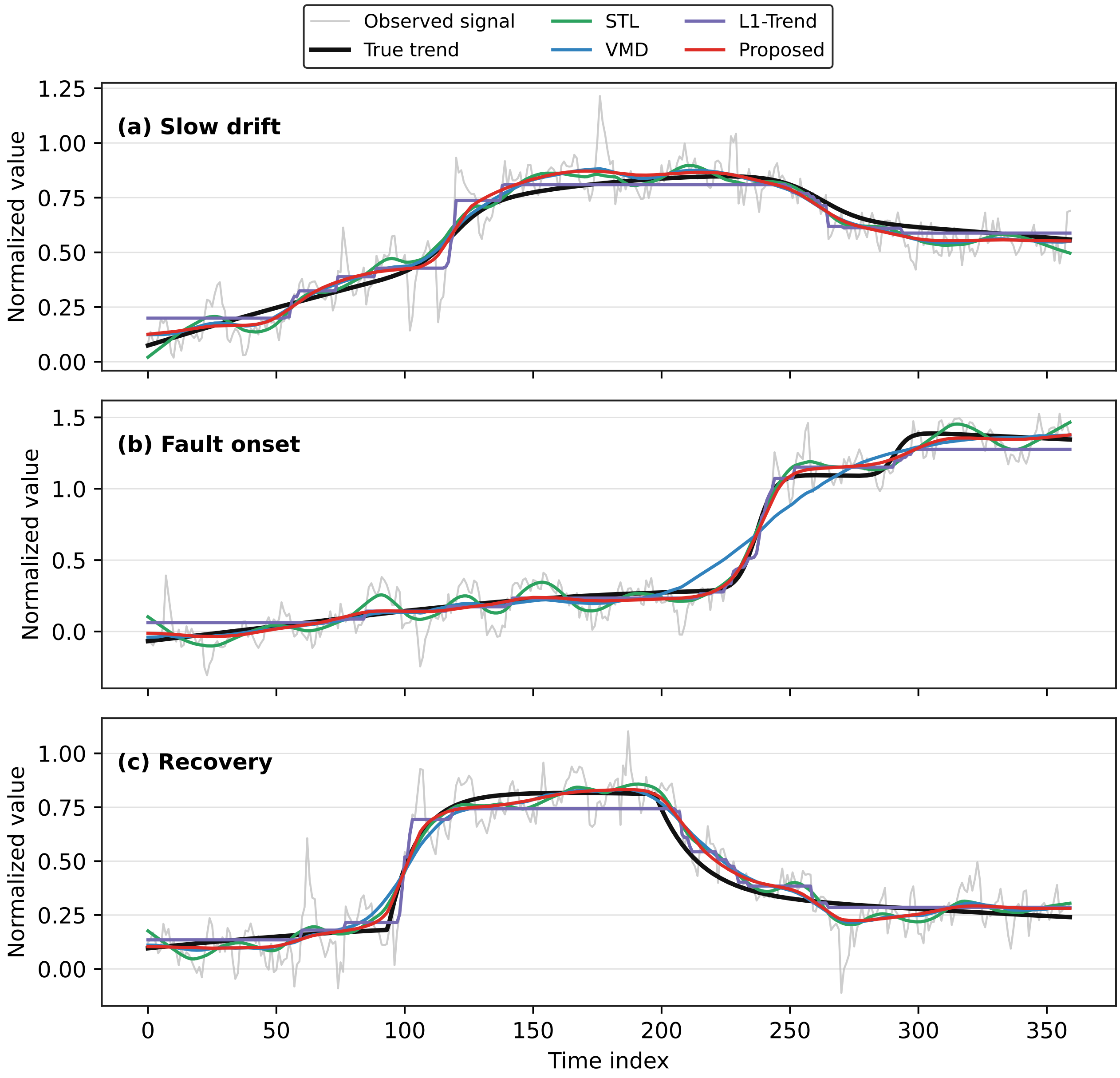

Figure S2: Controlled synthetic decomposition validation under slow drift, abrupt degradation-like transition, and recovery-like trend reversal. The black curve denotes the known macroscopic trend used for data generation.

S5 Extended Evaluation of M-Linear Trend Forecasting

Extending the forecasting examples shown in Section 4.2 of the main text, which highlight 4μm and viscosity, Figure S3 illustrates the multivariate trend-forecasting trajectories for the additional seven sensor channels in a single-column layout. Table S5 reports the complete predictive performance metrics.

Table S5: Detailed trend-forecasting performance metrics for individual sensors; FLOPs denote floating-point operations. The best results are **bolded**, and the second-best results are $\underline{\text{underlined}}$.

Sensor	Model	MAE	RMSE	R^2	Latency (ms)	Params (M)	FLOPs (G)
Density	**Ours**	2.35×10^{-4}	3.03×10^{-4}	0.991	**0.103**	$\underline{3.15 \times 10^{-4}}$	$\underline{9.30 \times 10^{-5}}$
	LSTM	2.18×10^{-3}	2.48×10^{-3}	0.377	$\underline{0.144}$	1.98×10^{-2}	8.98×10^{-4}
	DLinear	$\underline{9.96 \times 10^{-5}}$	$\underline{2.50 \times 10^{-4}}$	$\underline{0.994}$	0.517	$\mathbf{2.25 \times 10^{-4}}$	**0.00**
	iTransformer	1.40×10^{-3}	1.53×10^{-3}	0.765	0.327	6.86×10^{-2}	1.95×10^{-3}
	PatchTST	2.88×10^{-4}	5.13×10^{-4}	0.974	0.380	1.38×10^{-1}	1.20×10^{-3}
	ARIMA	$\mathbf{7.48 \times 10^{-6}}$	$\mathbf{1.54 \times 10^{-4}}$	**0.998**	0.990	-	-
Viscosity	**Ours**	$\underline{4.65 \times 10^{-2}}$	$\underline{2.48 \times 10^{-1}}$	$\underline{0.995}$	**0.103**	$\underline{3.15 \times 10^{-4}}$	$\underline{9.30 \times 10^{-5}}$
	LSTM	1.98	2.08	0.633	$\underline{0.144}$	1.98×10^{-2}	8.98×10^{-4}
	DLinear	5.12×10^{-2}	3.73×10^{-1}	0.988	0.517	$\mathbf{2.25 \times 10^{-4}}$	**0.00**
	iTransformer	1.63	1.78	0.730	0.327	6.86×10^{-2}	1.95×10^{-3}
	PatchTST	3.14×10^{-1}	5.76×10^{-1}	0.972	0.380	1.38×10^{-1}	1.20×10^{-3}
	ARIMA	$\mathbf{6.11 \times 10^{-3}}$	$\mathbf{1.83 \times 10^{-1}}$	**0.997**	0.990	-	-
Dielectric	**Ours**	$\underline{1.36 \times 10^{-4}}$	$\underline{5.12 \times 10^{-4}}$	$\underline{0.998}$	**0.103**	$\underline{3.15 \times 10^{-4}}$	$\underline{9.30 \times 10^{-5}}$
	LSTM	2.69×10^{-3}	3.34×10^{-3}	0.892	$\underline{0.144}$	1.98×10^{-2}	8.98×10^{-4}
	DLinear	1.78×10^{-4}	8.22×10^{-4}	0.993	0.517	$\mathbf{2.25 \times 10^{-4}}$	**0.00**
	iTransformer	8.07×10^{-3}	1.34×10^{-2}	-0.735	0.327	6.86×10^{-2}	1.95×10^{-3}
	PatchTST	8.94×10^{-4}	1.33×10^{-3}	0.983	0.380	1.38×10^{-1}	1.20×10^{-3}
	ARIMA	$\mathbf{1.61 \times 10^{-5}}$	$\mathbf{3.84 \times 10^{-4}}$	**0.999**	0.990	-	-
Temperature	**Ours**	$\underline{6.07 \times 10^{-2}}$	$\underline{2.47 \times 10^{-1}}$	$\underline{0.996}$	**0.103**	$\underline{3.15 \times 10^{-4}}$	$\underline{9.30 \times 10^{-5}}$
	LSTM	2.22	2.41	0.605	$\underline{0.144}$	1.98×10^{-2}	8.98×10^{-4}
	DLinear	7.42×10^{-2}	4.13×10^{-1}	0.988	0.517	$\mathbf{2.25 \times 10^{-4}}$	**0.00**
	iTransformer	4.71	5.70	-1.21	0.327	6.86×10^{-2}	1.95×10^{-3}
	PatchTST	8.98×10^{-1}	4.09	-0.136	0.380	1.38×10^{-1}	1.20×10^{-3}
	ARIMA	$\mathbf{7.19 \times 10^{-3}}$	$\mathbf{1.87 \times 10^{-1}}$	**0.998**	0.990	-	-
4μm	**Ours**	$\underline{10.7}$	$\underline{43.7}$	$\underline{0.992}$	**0.103**	$\underline{3.15 \times 10^{-4}}$	$\underline{9.30 \times 10^{-5}}$
	LSTM	91.4	152	0.908	$\underline{0.144}$	1.98×10^{-2}	8.98×10^{-4}
	DLinear	24.9	60.3	0.986	0.517	$\mathbf{2.25 \times 10^{-4}}$	**0.00**
	iTransformer	101	232	0.785	0.327	6.86×10^{-2}	1.95×10^{-3}
	PatchTST	42.1	255	0.741	0.380	1.38×10^{-1}	1.20×10^{-3}
	ARIMA	**2.26**	**27.5**	**0.997**	0.990	-	-
6μm	**Ours**	$\underline{3.87}$	$\underline{20.5}$	$\underline{0.987}$	**0.103**	$\underline{3.15 \times 10^{-4}}$	$\underline{9.30 \times 10^{-5}}$
	LSTM	54.8	73.2	0.831	$\underline{0.144}$	1.98×10^{-2}	8.98×10^{-4}
	DLinear	6.29	23.9	0.982	0.517	$\mathbf{2.25 \times 10^{-4}}$	**0.00**
	iTransformer	49.4	97.7	0.700	0.327	6.86×10^{-2}	1.95×10^{-3}
	PatchTST	10.9	48.5	0.926	0.380	1.38×10^{-1}	1.20×10^{-3}
	ARIMA	**1.09**	**16.6**	**0.991**	0.990	-	-
14μm	**Ours**	2.62	15.4	$\underline{0.982}$	**0.103**	$\underline{3.15 \times 10^{-4}}$	$\underline{9.30 \times 10^{-5}}$
	LSTM	45.4	65.8	0.667	$\underline{0.144}$	1.98×10^{-2}	8.98×10^{-4}
	DLinear	$\underline{2.36}$	$\underline{15.2}$	$\underline{0.982}$	0.517	$\mathbf{2.25 \times 10^{-4}}$	**0.00**
	iTransformer	28.6	44.6	0.847	0.327	6.86×10^{-2}	1.95×10^{-3}
	PatchTST	7.59	39.3	0.881	0.380	1.38×10^{-1}	1.20×10^{-3}
	ARIMA	**0.812**	**12.9**	**0.987**	0.990	-	-
21μm	**Ours**	$\underline{2.42}$	$\underline{14.3}$	$\underline{0.981}$	**0.103**	$\underline{3.15 \times 10^{-4}}$	$\underline{9.30 \times 10^{-5}}$
	LSTM	44.4	64.5	0.611	$\underline{0.144}$	1.98×10^{-2}	8.98×10^{-4}
	DLinear	4.52	14.9	0.979	0.517	$\mathbf{2.25 \times 10^{-4}}$	**0.00**
	iTransformer	25.8	37.7	0.867	0.327	6.86×10^{-2}	1.95×10^{-3}
	PatchTST	6.98	36.2	0.878	0.380	1.38×10^{-1}	1.20×10^{-3}
	ARIMA	**0.756**	**11.9**	**0.987**	0.990	-	-
Water Content	**Ours**	$\underline{2.02 \times 10^{-2}}$	$\underline{5.06 \times 10^{-2}}$	$\underline{0.999}$	**0.103**	$\underline{3.15 \times 10^{-4}}$	$\underline{9.30 \times 10^{-5}}$
	LSTM	2.26	2.45	-0.636	$\underline{0.144}$	1.98×10^{-2}	8.98×10^{-4}
	DLinear	8.36×10^{-2}	1.06×10^{-1}	0.997	0.517	$\mathbf{2.25 \times 10^{-4}}$	**0.00**
	iTransformer	2.67	3.27	-1.90	0.327	6.86×10^{-2}	1.95×10^{-3}
	PatchTST	6.77×10^{-1}	9.30×10^{-1}	0.765	0.380	1.38×10^{-1}	1.20×10^{-3}
	ARIMA	$\mathbf{2.28 \times 10^{-3}}$	$\mathbf{2.37 \times 10^{-2}}$	**1.00**	0.990	-	-

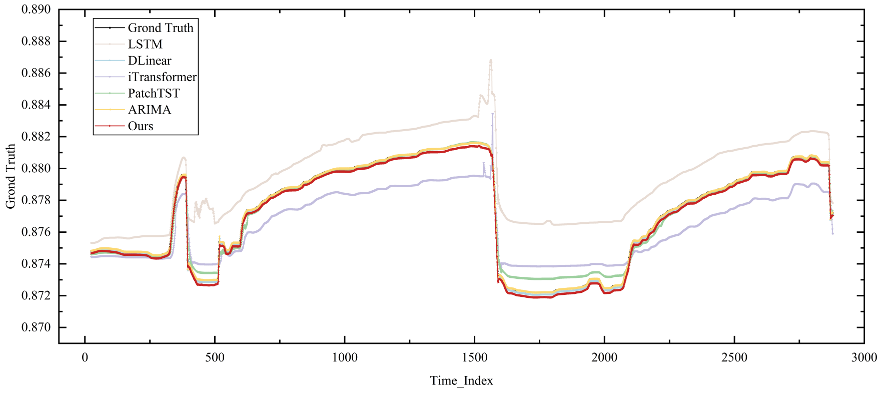

(a) Density

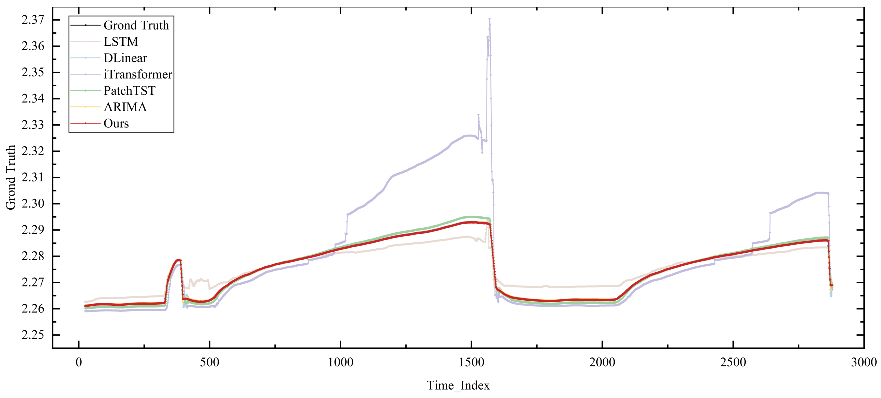

(b) Dielectric Constant

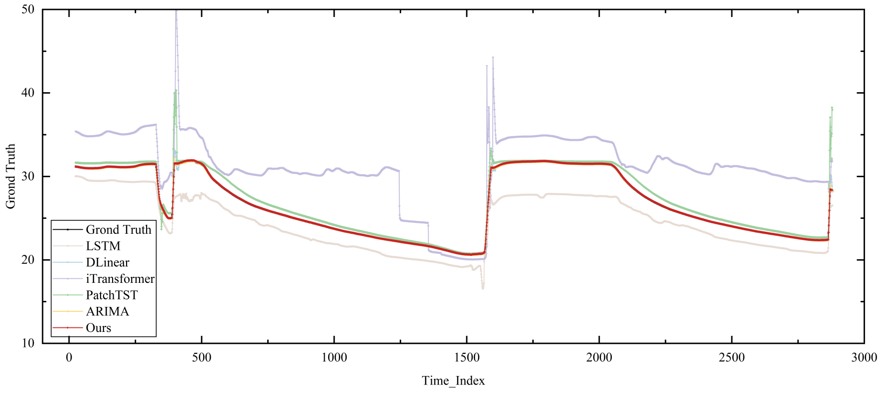

(c) Temperature

Figure S3: Comparative results of multivariate trend forecasting on extended parameters.

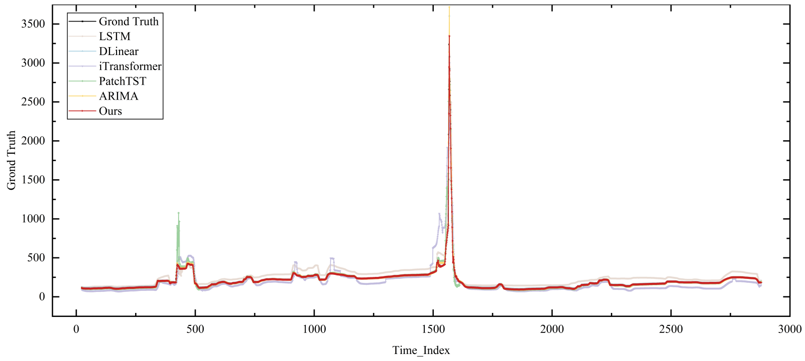

(d) 6μm

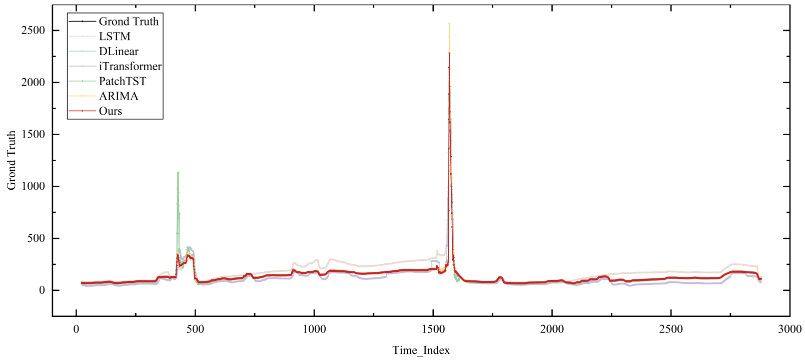

(e) 14μm

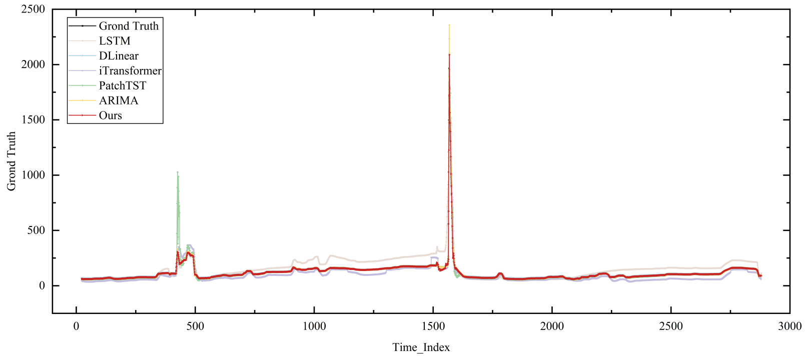

(f) 21μm

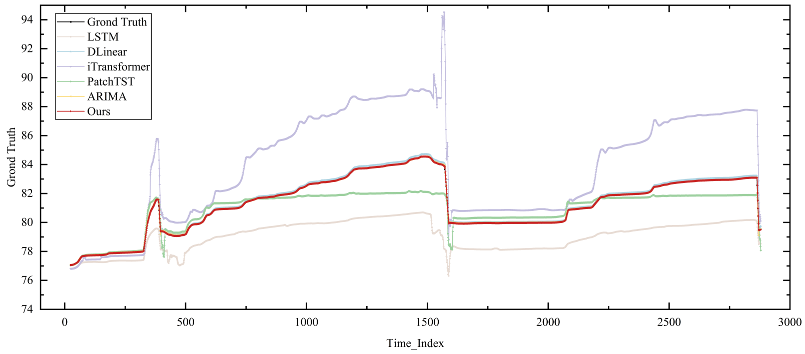

(g) Moisture (Water Content)

Figure S3: Comparative results of multivariate trend forecasting on extended parameters (cont.).

S6 Residual Distribution Diagnostics

To complement the residual distribution diagnostics reported in the main manuscript, this section provides the complete sensor-wise residual statistics and additional residual diagnostic figures. Table S6 summarizes the training-interval residual skewness, excess kurtosis, Jarque–Bera normality-test results, and the coefficient of variation (CV) of rolling residual standard deviation. Figure S4 shows residual distribution diagnostics for the remaining monitored parameters, and Figure S5 presents the test-time evolution of residual volatility and skewness across all nine sensors.

Table S6: Residual distribution statistics for individual sensors in the training interval.

**Sensor**	**Skewness**	**Excess Kurtosis**	**JB p-value**	**Rolling Std. CV**
Density	-0.08	2.49	$p < 0.001$	0.34
Viscosity	0.33	2.33	$p < 0.001$	0.36
Dielectric Constant	-0.38	1.01	$p < 0.001$	0.19
Temperature	0.82	14.96	$p < 0.001$	0.67
4μm	2.66	45.01	$p < 0.001$	0.75
6μm	2.49	24.59	$p < 0.001$	0.72
14μm	2.43	25.24	$p < 0.001$	0.66
21μm	2.32	25.83	$p < 0.001$	0.65
Water Content	-2.39	10.32	$p < 0.001$	0.47

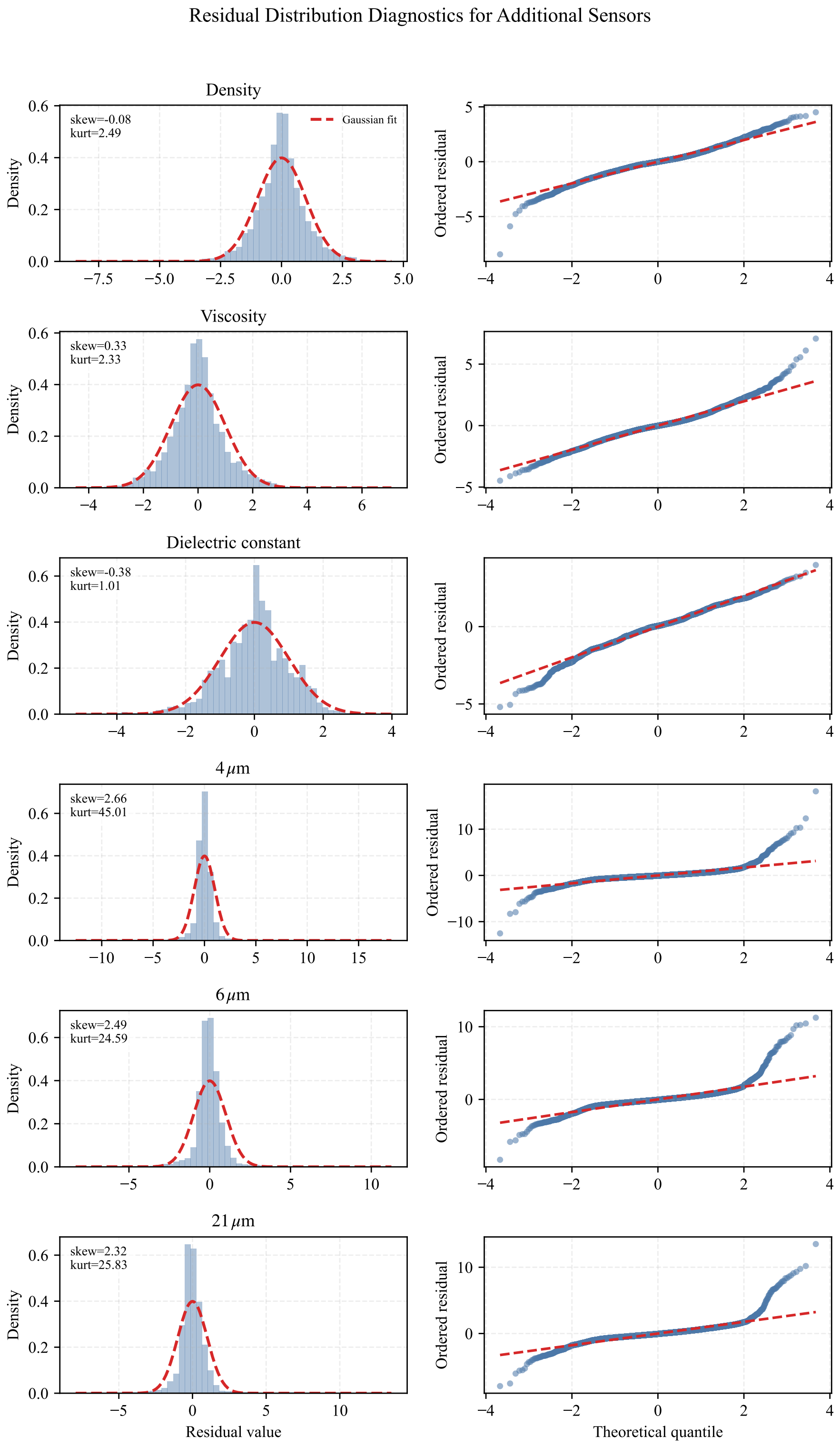

Figure S4: Residual distribution diagnostics for the remaining monitored parameters in the training interval, including empirical residual distributions and Gaussian-reference Q-Q diagnostics.

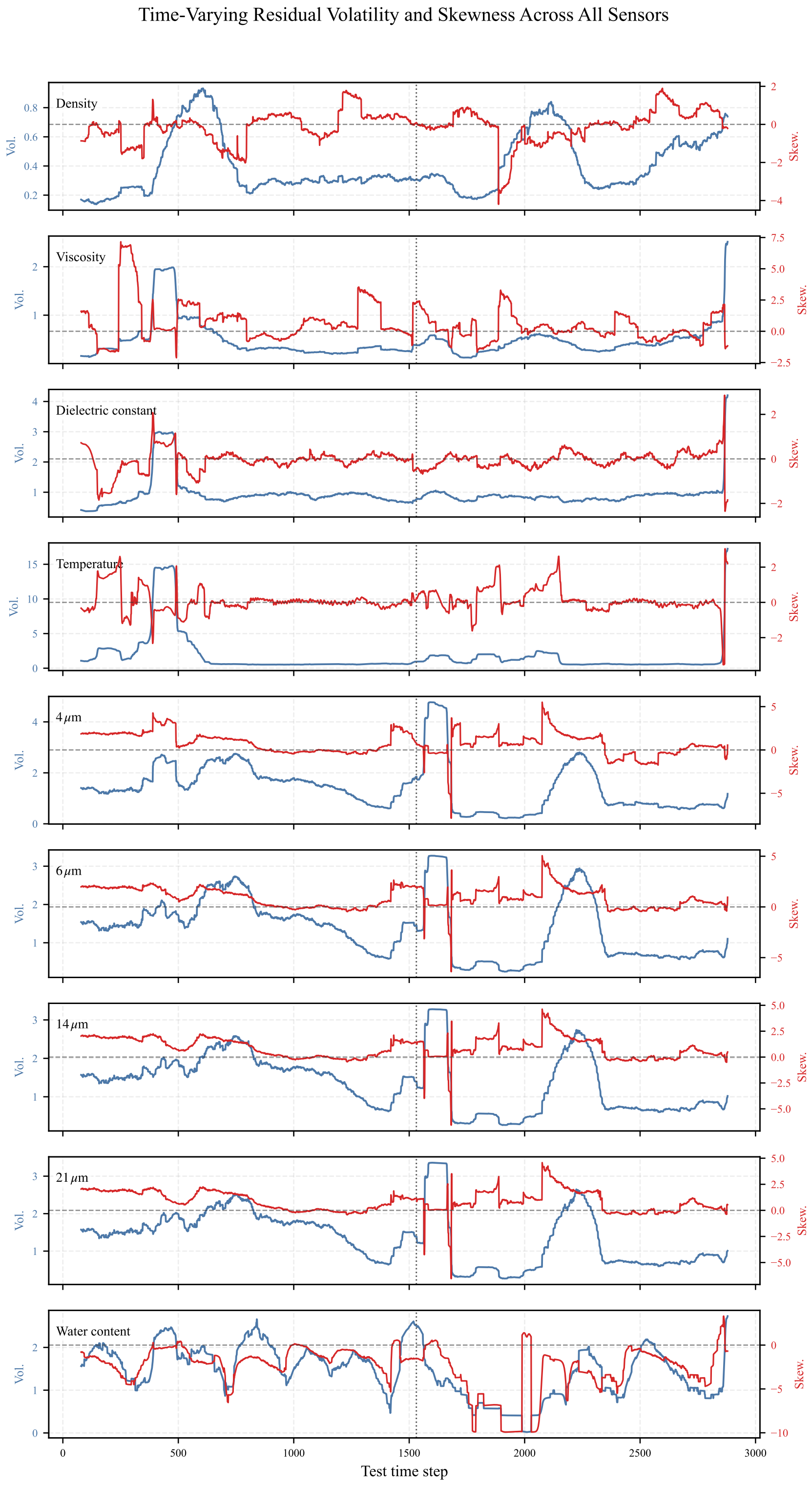

Figure S5: Test-time evolution of residual volatility and skewness across all monitored parameters, summarizing sensor-wise residual-distribution dynamics.
